# Assessing thyroid health: phenotypic age compared to chronological age

**DOI:** 10.3389/fendo.2025.1594139

**Published:** 2025-07-04

**Authors:** Dongyu Yang, Cihang Lu, Haonan Zhang, Xiaoguang Shi, Ying Sun, Ying Shao, Shuting Fan, Lijun Tian, Di Teng

**Affiliations:** ^1^ The Department of Endocrinology, Shengjing Hospital of China Medical University, Shenyang, Liaoning, China; ^2^ The Department of Endocrinology and Metabolism, Institute of Endocrinology, National Health Commission Key Laboratory of Diagnosis and Treatment of Thyroid Diseases, The First Hospital of China Medical University, Shenyang, Liaoning, China

**Keywords:** phenotypic age, aging, hypothyroidism, hyperthyroidism, thyroid antibody, FT3, FT4, TSH

## Abstract

**Introduction:**

Aging is associated with thyroid dysfunction, but the role of phenotypic age, a biological aging measure derived from nine clinical biomarkers and chronological age, remains unclear.

**Methods:**

This cross-sectional study included 6,681 adults from the National Health and Nutrition Examination Survey (NHANES, 2007–2012) with complete thyroid function and age data. Participants were grouped into quartiles based on chronological and phenotypic age. Weighted multinomial logistic regression was used to assess the association between aging and thyroid disorders, followed by the use of restricted cubic splines (RCSs) to explore potential nonlinear relationships. Subgroup and sensitivity analyses were conducted to test robustness. Mediation analysis assessed the role of phenotypic age components in the link between phenotypic age and thyroid dysfunction.

**Results:**

Thyroid-stimulating hormone (TSH) and free thyroxine (FT4) exhibited U-shaped relationships with both chronological and phenotypic age, while free triiodothyronine (FT3) showed a nonlinear association with chronological age and a negative linear correlation with phenotypic age. The age gap (phenotypic age minus chronological age) was positively associated with TSH and nonlinearly with FT4. Thyroid peroxidase antibody (TPOAb) exhibits a nonlinear association with both age types, and thyroglobulin antibody (TGAb) has a positive linear association with chronological age. PPhenotypic age showed stronger linear associations with TPOAb positivity (PTPOAb), TGAb positivity (PTGAb), overt hyperthyroidism, and subclinical hypothyroidism than chronological age. Overt hypothyroidism demonstrated an inverted U-shaped association with both age metrics and a positive correlation with age gap. Mediation analysis revealed that mean cell volume mediated 10% of the association between phenotypic age and overt hypothyroidism, while lymphocyte percentage exhibited a negative mediation effect (−26%) in the association between phenotypic age and subclinical hypothyroidism.

**Discussion:**

Phenotypic age better captures aging-related changes in thyroid function than chronological age and may serve as a useful biological aging marker in clinical endocrine research.

## Introduction

Thyroid hormones play a vital role in regulating growth and metabolism. Abnormal levels are characteristic of conditions such as hyperthyroidism and hypothyroidism, which may result from underlying causes including autoimmune thyroiditis (AIT) (1, 2). In iodine-sufficient regions, hyperthyroidism affects about 0.2–1.3% of the population (3), typically presents with symptoms such as nervousness, heat intolerance, and weight loss. Severe cases can progress to thyroid storm, a life-threatening condition marked by dangerously high heart rate, fever, and mental confusion, which requires urgent medical intervention (1, 4). Hypothyroidism, on the other hand, ranges from asymptomatic to severe cases, such as myxedema coma. Common adult symptoms include fatigue, cold intolerance, weight gain, and dry skin. Its prevalence is approximately 0.2%–5.3% in Europe (5) and 4.3% in the U.S (6). Thyroid autoimmunity markers include thyroglobulin antibodies (TGAb) and thyroid peroxidase antibodies (TPOAb), with TPOAb present in about 11% of the population and linked to progression from subclinical to overt hypothyroidism (6, 7). Given the nonspecific symptoms of thyroid dysfunction (e.g., fatigue, cold intolerance), thyroid function testing is widely utilized for diagnosis. A confirmed diagnosis typically requires long-term management, highlighting the clinical significance of accurate testing (1, 2, 8).

The effect of chronological age on thyroid-stimulating hormone (TSH), free triiodothyronine (FT3), and free thyroxine (FT4) levels varies across studies. While most studies suggest that TSH levels tend to increase with age in adults (9–13), some report a decrease (14, 15) or no significant change (16–18), and a few indicate a U-shaped distribution (19). The associations between age and FT3 (10, 13, 18) or FT4 (9, 10, 13, 16, 17) levels are similarly inconclusive, indicating the need for further research.

Aging is a multifaceted and heterogeneous process that progressively erodes the structural integrity and functional resilience of cells, tissues, and organs (20). While chronological age simply reflects the passage of time, it often fails to capture the significant inter-individual variability in physiological decline and disease susceptibility. Consequently, a more sophisticated measure, biological age, has emerged to quantify an individual’s true physiological state, reflecting accumulated molecular and cellular damage rather than mere temporal progression. Biological aging indicators, such as phenotypic age, synthesize a range of objective biological markers into a composite score, providing a more precise assessment of an individual’s ‘functional age’ or physiological burden. With the advancement of measurement technologies, molecular epidemiology now incorporates various aspects of aging, allowing for a more comprehensive understanding of the aging process (21, 22). The current metrics in aging biology can be categorized into high-dimensional ‘clocks’ derived from omics data—such as DNA methylation, transcriptomics, metabolomics, and proteomics—and those based on conventional clinical biomarkers. The plasma proteomic aging clock, based on the Gompertz mortality model, is one such phenotypic age measure (23). It is derived from nine specific clinical biomarkers alongside chronological age, and performs well in capturing morbidity and mortality in multiple populations (24, 25).

Furthermore, the phenotypic age that comprise clinical biochemical indicators are not only more readily available but also commonly measured in routine health check-ups, which makes phenotypic age a practical and cost-effective tool for assessing the biological aging process. Despite its advantages, the specific relationship between phenotypic age and thyroid function indicators remains unclear.

In this study, we use cross-sectional data from the National Health and Nutrition Examination Survey (NHANES) database to evaluate the associations of chronological and phenotypic age with thyroid function indicators and thyroid dysfunction diseases. Weighted multinomial logistic regression models are applied to analyze these associations, and restricted cubic splines (RCSs) are used to explore potential nonlinear relationships. To ensure the robustness of our findings, we further conduct subgroup and sensitivity analyses.

## Materials and methods

### Study population

Data from the NHANES database, spanning the survey cycles of 2007-2008, 2009-2010, and 2011-2012, was used in this study. The NHANES employs a multistage, stratified sampling method to achieve a representative sample of the U.S. civilian, non-institutionalized population. Participants received comprehensive home visits and physical examinations to assess their health and nutritional status. The study received ethical approval from the Institutional Review Board of the National Center for Health Statistics (NCHS), with informed consent obtained from all participants (https://www.cdc.gov/nchs/nhanes/irba98.htm). Detailed information on the survey’s methodology, sampling procedures, and laboratory tests can be found on the CDC’s official website (http://www.cdc.gov/nchs/nhanes).

Initially, there were 30,442 individuals in the NHANES 2007–2012 dataset. Exclusions were made for individuals who: 1) were younger than 18 years (n = 11,823), 2) had a history of thyroid disease (n = 667) as reported in their medical condition questionnaire, 3) had incomplete information on thyroid function indicators (n = 9,412), 4) had incomplete thyroid antibody indicators information (n = 133), and 5) had incomplete phenotypic indicators information (n = 1,726). Consequently, the final analysis sample included 6,681 unique participants. After further excluding those with missing covariates, the total number of participants in the sensitivity analysis was 5,089. The participant flow is shown in **Supplementary Figure S1**.

### Diagnostic criteria

To determine thyroid function, participants were measured for thyroid stimulating hormone (TSH), free thyroxine (FT4), total thyroxine (TT4), free triiodothyronine (FT3), total triiodothyronine (TT3), thyroid peroxidase antibody (TPOAb), and thyroglobulin antibody (TGAb). The concentrations of FT3, TT3, and TT4 were assessed using competitive binding immunoenzymatic assays. FT4 was measured through a two-step enzyme immunoassay, while TSH was determined using a third-generation two-site immunoenzymatic assay. TPOAb and TGAb titers were evaluated with the Beckman Access2 immunoassay system.

Various thyroid disorders, including overt hyperthyroidism, subclinical hyperthyroidism, overt hypothyroidism, subclinical hypothyroidism, and autoimmune thyroiditis (AIT), were diagnosed based on specific criteria. The normal ranges for thyroid function indicators were as follows: TSH (0.34–5.6 mIU/L), FT4 (7.74–20.6 pmol/L), TT4 (4.5–13.2 µg/dL), FT3 (2.5–3.9 pg/mL), TT3 (87–178 ng/dL). Subclinical hyperthyroidism was defined by TSH < 0.34 mIU/L with FT3 and FT4 within normal range. Overt hyperthyroidism was characterized by TSH < 0.34 mIU/L and FT4 > 20.6 pmol/L. Subclinical hypothyroidism was indicated by TSH > 5.6 mIU/L with FT4 within normal range, while overt hypothyroidism was marked by TSH > 5.6 mIU/L and FT4 < 7.74 pmol/L. Thyroid antibody positivity was categorized into positive and negative groups: TPOAb positive (PTPOAb) (> 34 IU/mL), and TGAb positive (PTGAb) (> 4.0 IU/mL).

### Methods for calculating phenotypic age

The calculation of phenotypic age is based on a combination of clinical biomarkers and chronological age, using Cox proportional hazards and Gompertz models to assess aging-related mortality risks. The data informing this method comes from the National Health and Nutrition Examination Survey (NHANES). Specifically, the models used aim to predict a 10-year mortality risk by evaluating the effects of both age and biomarkers on survival (23, 26). Ten specific biomarkers were selected for the final model:Albumin (ALB, g/L), Creatinine (CR, μmol/L), Glucose (GLU, mmol/L), C-reactive protein (CRP, mg/dL), Lymphocyte percentage (L%, %), Mean cell volume (MCV, fL), Red cell distribution width (RDW, %), Alkaline phosphatase (ALP, U/L), White blood cell count (WBC, 10^9/L), Chronological age. These nine clinical chemistry biomarkers collectively represent key physiological systems and metabolic pathways crucial for overall health and aging. Specifically, ALB and ALP are indicators of liver function; CR reflects kidney function; GLU assesses metabolic status; CRP signifies inflammation; and L%, MCV, RDW, and WBC reflect immune and hematological health. Perturbations in these fundamental physiological processes are frequently interconnected with endocrine homeostasis, including thyroid function.

In addition, we calculated the difference between phenotypic age and chronological age to obtain the age gap. The age gap was used to determine whether an individual’s phenotypic age was younger or older than their chronological age; a negative age gap indicated a younger phenotypic age.

### Statistical analysis

The “survey” “nhanesR” “rms” “mice”package in R was utilized for the complex weighting of the analysis, applying NHANES-specific MCE weights to ensure that the findings accurately represent the population. Data sets were merged according to the unique participant identifier, SEQN, in accordance with NHANES guidelines. A check for duplicate values on SEQN was conducted to ensure that no participant was inadvertently included more than once. Additional details on data merging can be found on the CDC website. (https://www.cdc.gov/nchs/tutorials/nhanes-cms/determine/merge.html). The “mice” package in R was used for multiple imputation to address missing data. Comprehensive details on missing values, including the variables and the number of missing entries, are provided in **Supplementary Table S1**.

Normality of data was first analyzed using the Kolmogorov-Smirnov test. Populations were divided into four groups based on quartiles of their chronological and phenotypic ages. Categorical variables were expressed as numbers (percentages) and compared using the Rao-Scott Chi-Square test. Continuous variables were expressed as means with 95% confidence intervals (mean ± 95% CI) and compared using ANOVA based on Taylor Series Linearization (TSL).

Weighted logistic regression models were used to investigate the association between chronological, phenotypic age and thyroid dysfunction, with the lowest age group (Q1) as the reference category. Model 1 was unadjusted for any covariates. Model 2 included adjustments for sex, age, BMI, race/ethnicity, income, education level, marital status, smoking status, drinking status, physical activity level, and healthy eating score. Model 3 incorporated further adjustments for self-reported health status (very good to excellent, good, poor to fair) and self-reported disease status, including cardiovascular disease, neoplasms, and Chronic obstructive pulmonary disease.

To ensure the robustness of the findings, sensitivity analysis was conducted by excluding participants with incomplete covariate data. Nonlinear relationships were evaluated using restricted cubic splines (RCSs), adjusted for the same covariates as in the regression models. The selection of knots aims to minimize the difference between the model’s predicted values and the actual observed values, that is, to achieve the smallest residual at specific knot locations. Subgroup analysis were performed across various demographic and lifestyle factors. Mediation analysis were performed using the “mediation” package in R statistical software.

## Results

### Subjects characteristics

The study included 6,681 participants (mean age: 44.79 years, 95% CI: 44.00–45.58) (**Table 1**), with 52.46% men (n = 3,534) and 47.54% women (n = 3,147). Participants in the highest chronological age quartile (Q4) were older (mean age: 70.12 years), predominantly non-Hispanic White (75.52%), and had the lowest proportion of individuals with a college education or above (41.71%). This group had a mean BMI of 29.7 kg/m², a urine iodine concentration of 366.9 µg/L, lower smoking prevalence (13.25%), and higher rates of no leisure-time physical activity (64.58%). **Supplementary Figure S2** illustrates a strong positive linear correlation between phenotypic and chronological age (P < 0.001, P for Non-linearity = 0.246), confirming their close association. **Supplementary Figure S3** shows that most individuals had a negative age gap, suggesting a younger phenotypic age. Further details on thyroid indicators and the prevalence of thyroid diseases across chronological age, phenotypic age, and age gap quartiles are provided in **Supplementary Tables S2–S4**, respectively.

**Table 1:** Baseline characteristics of American adults stratified by age quartiles, NHANES 2007-2012.

Characteristic	Total	Quartile 1	Quartile 2	Quartile 3	Quartile 4	*P* value
Participants (%)	6681 (100%)	1671 (25.01%)	1669 (24.98%)	1671 (25.01%)	1670 (25.00%)	
Age (yr)^a^ BMI (kg/m^2^)^a^ UIC (ug/L) ^a^	44.79 (44.00 - 45.58) 28.31 (28.06 - 28.57) 254.93 (227.45 - 282.40)	25.82 (25.46 - 26.18) 26.12 (25.68 - 26.56) 225.54 (180.54 - 270.53)	40.51 (40.05 - 40.98) 28.74 (28.28 - 29.20) 208.58 (185.81 - 231.32)	54.37 (53.94 - 54.80) 29.33 (28.98 - 29.67) 269.77 (184.50 - 355.05)	70.12 (69.47 - 70.78) 29.70 (29.26 - 30.15) 366.86 (298.37 - 435.36)	< 0.0001 < 0.0001 < 0.001
Sex (%)^b^						0.63
Male	3534 (52.46%)	862 (52.14%)	827 (51.38%)	874 (52.93%)	971 (54.22%)	
Female	3147 (47.54%)	809 (47.86%)	842 (48.62%)	797 (47.07%)	699 (45.78%)	
Social and economic parameters (%)^b^						
Race/ethnicity						< 0.0001
Non-Hispanic white	3069 (68.31%)	657 (62.08%)	753 (66.74%)	752 (72.31%)	907 (75.52%)	
Non-Hispanic black	1293 (10.84)	325 (11.42%)	271 (9.79%)	348 (10.95%)	349 (11.59%)	
Mexican American	1225 ( 8.91)	388 (12.56%)	352 (10.49%)	282 (5.68%)	203 (4.96%)	
Others	1094 (11.93)	301 (13.95%)	293 (12.97%)	289 (11.07%)	211 (7.93%)	
Education status						< 0.0001
Less than high school	2048 (20.54%)	442 (18.65%)	477 (20.16%)	472 (17.10%)	657 (30.21%)	
High school or equivalent	1593 (24.50%)	395 (22.75%)	378 (21.84%)	418 (27.13%)	402 (28.07%)	
College or above	3040 (54.96%)	834 (58.60%)	814 (57.99%)	781 (55.77%)	611 (41.71%)	
Family income-poverty ratio level						< 0.0001
0-1.0	1489 (15.10%)	489 (21.17%)	381 (14.84%)	306 (10.26%)	313 (12.87%)	
1.1-3.0	2904 (36.48%)	695 (37.70%)	662 (32.60%)	653 (30.67%)	894 (51.01%)	
>3.0	2288 (48.42%)	487 (41.13%)	626 (52.56%)	712 (59.07%)	463 (36.12%)	
Marital status						< 0.0001
Married	3961 (63.19%)	764 (48.16%)	1149 (71.93%)	1110 (71.88%)	938 (59.24%)	
Separated	1411 (17.09%)	87 (4.91%)	263 (14.94%)	421 (20.71%)	640 (36.42%)	
Never married	1309 (19.72%)	820 (46.93%)	257 (13.14%)	140 (7.41%)	92 (4.34%)	
Smoking status						< 0.0001
Never smoker	3553 (53.67%)	1069 (60.80%)	950 (57.60%)	804 (47.61%)	730 (43.94%)	
Former smoker	1618 (23.66%)	180 (13.21%)	286 (18.95%)	453 (28.39%)	699 (42.81%)	
Current smoke	1510 (22.67%)	422 (25.99%)	433 (23.45%)	414 (24.00%)	241 (13.25%)	
Drinking status						< 0.0001
Non-drinker	1731 (22.17%)	353 (18.16%)	371 (17.51%)	443 (24.27%)	564 (34.26%)	
Low - moderate drinker	3932 (63.68%)	1140 (71.92%)	1026 (66.26%)	937 (59.47%)	829 (51.45%)	
Heavy drinker	1018 (14.15%)	178 (9.92%)	272 (16.23%)	291 (16.26%)	277 (14.29%)	
Healthy eating index score						< 0.0001
Quartile 1	1844 (28.20%)	557 (33.49%)	520 (31.09%)	423 (24.47%)	344 (19.76%)	
Quartile 2	1724 (25.95%)	486 (27.26%)	435 (26.06%)	402 (25.41%)	401 (24.33%)	
Quartile 3	1808 (26.72%)	419 (25.65%)	434 (25.41%)	496 (29.22%)	459 (26.84%)	
Quartile 4	1305 (19.14%)	209 (13.60%)	280 (17.44%)	350 (20.90%)	466 (29.07%)	
Leisure time physical activity level						< 0.0001
0 times/week	3646 (46.98%)	659 (33.98%)	850 (43.79%)	990 (53.66%)	1147 (64.58%)	
1–2 times/week	855 (15.49%)	296 (19.75%)	245 (16.96%)	185 (13.41%)	129 (8.74%)	
≥3 times/week	2180 (37.54%)	716 (46.27%)	574 (39.25%)	496 (32.93%)	394 (26.68%)	

BMI, Body Mass Index; UIC, Urine Iodine Concentration.

a presented as mean (95% confidence interval).

b presented as mean (frequency).

### Dose-response relationships of aging with thyroid function and diseases

**Figure 1:**
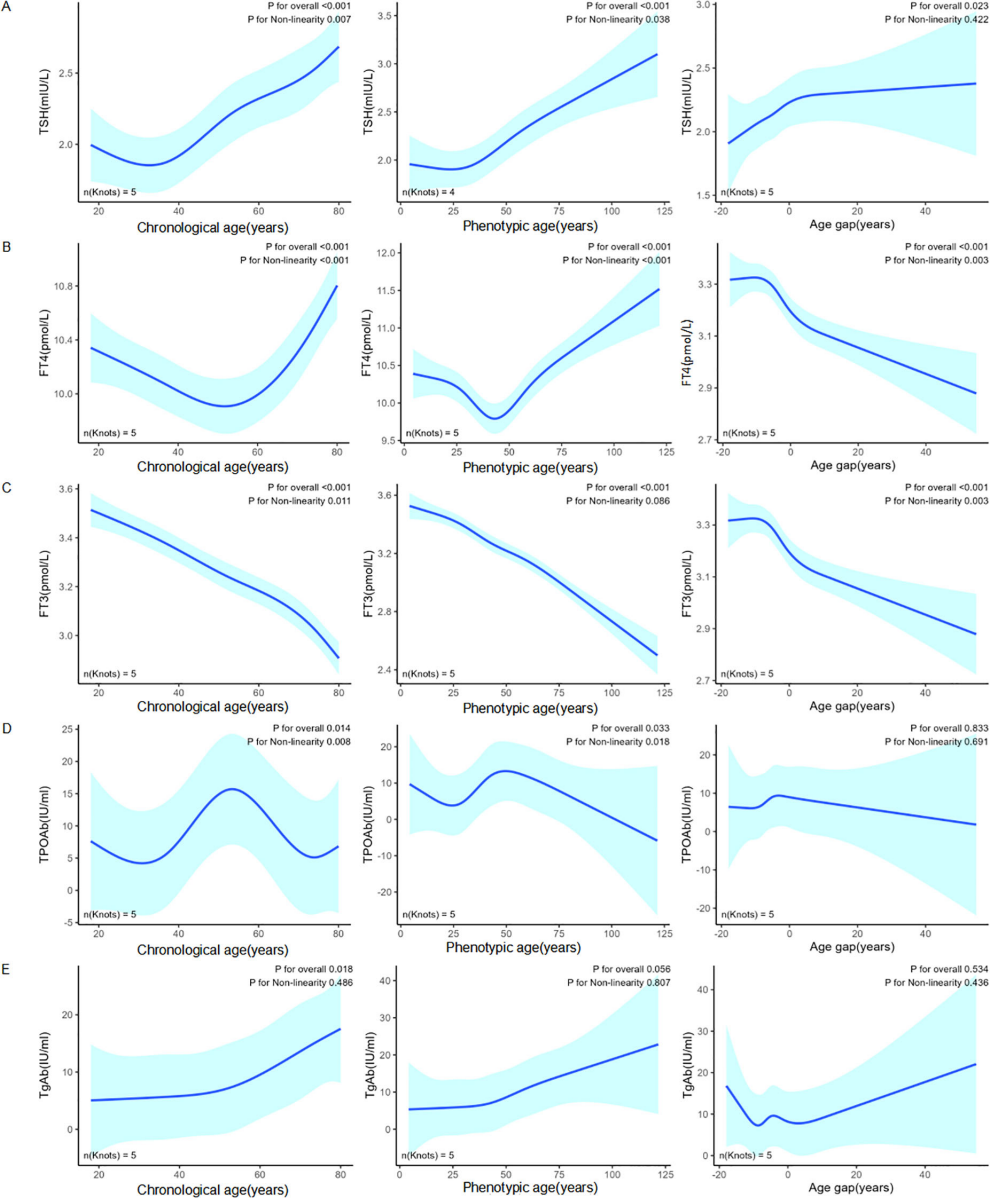
Restricted cubic spline (RCS) plots showing the associations between thyroid function markers and three age metrics: chronological age (left), phenotypic age (middle), and age gap (right). The blue lines represent predicted values from RCS models, with shaded areas indicating 95% confidence intervals. **(A)** TSH (mIU/L); **(B)** FT4 (pmol/L); **(C)** FT3 (pmol/L); **(D)** TPOAb (IU/mL); **(E)** TgAb (IU/mL).

Restricted cubic spline (RCS) analysis revealed several nonlinear associations between aging metrics and thyroid function indicators (**Figure 1**). TSH and FT4 both exhibited U-shaped relationships with chronological and phenotypic age, whereas FT3 showed a nonlinear decline with chronological age and a negative linear association with phenotypic age. Notably, TPOAb showed a nonlinear association with both age types, while TGAb displayed a positive linear association with chronological age. When evaluating age gap, TSH demonstrated a positive linear relationship, whereas FT4 exhibited a nonlinear pattern.

**Figure 2:**
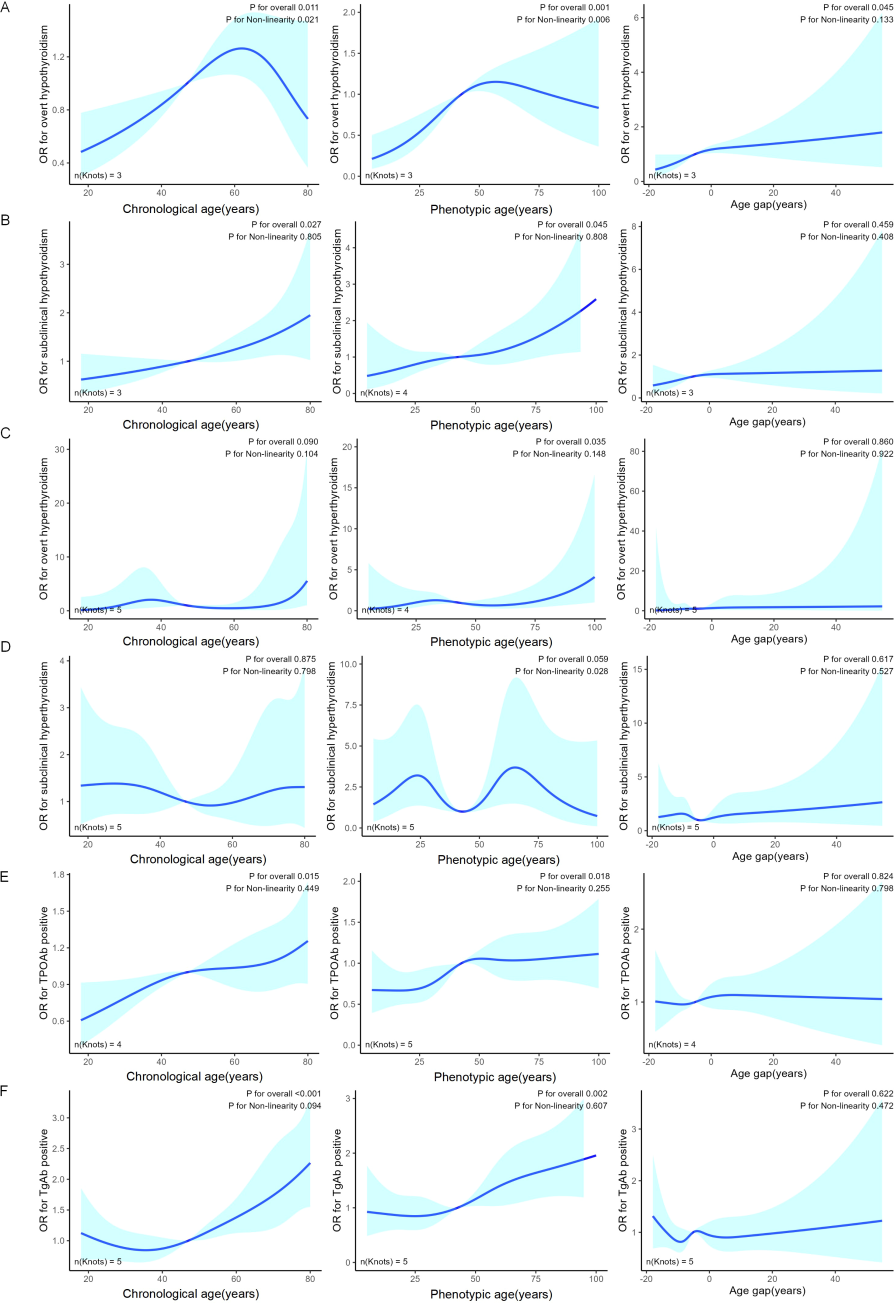
RCSs plots of thyroid dysfunction across chronological age, phenotypic age, and age gap. The plots depict the relationship between various thyroid dysfunctions and age, categorized as follows: Overt Hypothyroidism **(A)**, Subclinical Hypothyroidism **(B)**, Overt Hyperthyroidism **(C)**, Subclinical Hyperthyroidism **(D)**, TPOAb positive **(E)**, and TGAb positive **(F)**. Each plot presents data for chronological age (left), phenotypic age (middle), and age gap (right). The blue lines represent the predicted values obtained using RCS regression, while the shaded areas indicate the 95% confidence intervals.

For thyroid dysfunction (**Figure 2**), overt hypothyroidism exhibited an inverted U-shaped association with both chronological and phenotypic age and a positive linear association with age gap. Subclinical hypothyroidism and thyroid autoantibody positivity showed a positive linear relationship with both chronological and phenotypic age. Overt hyperthyroidism exhibited a linear increase with phenotypic age. These results suggest that phenotypic age more consistently captures aging-related changes in thyroid function compared to chronological age.

### Quantitative association between aging and thyroid disorders (Logistic Regression)

To support the nonlinear associations observed in RCS analysis, we conducted multinomial logistic regression to evaluate the odds of thyroid dysfunction across quartiles of chronological and phenotypic age (**Table 2**). Although some associations did not reach conventional levels of statistical significance, they revealed consistent directional trends.

**Table 2:** Multinomial logistic regression analysis for the relationships of aging with thyroid dysfunction.

Chronological Age				Phenotypic age			
Subclinical hypothyroidism				Subclinical hypothyroidism			
Model 1	P	OR (95% CI)	P trend	Model 1	P	OR (95% CI)	P trend
Q1 Q2 Q3 ** Q4**	Ref 0.57 0.17 **0.01**	Ref 1.20(0.63,2.29) 1.56(0.82,2.96) **2.34(1.27,4.30)**	**0.01**	Q1 **Q2** Q3 **Q4**	Ref **0.05** 0.18 **0.004**	Ref **1.78(1.01,3.15)** 1.61(0.79,3.28) **2.35(1.34,4.12)**	**0.02**
Model 2	P	OR (95% CI)	P trend	Model 2	P	OR (95% CI)	P trend
Q1 Q2 Q3 ** Q4**	Ref 0.61 0.24 **0.01**	Ref 1.20(0.58,2.46) 1.57(0.73,3.34) **2.25(1.19,4.24)**	**0.03**	Q1 **Q2** Q3 **Q4**	Ref **0.04** 0.18 **0.01**	Ref **1.83(1.04,3.24)** 1.68(0.78,3.63) **2.43(1.20,4.91)**	0.06
Model 3	P	OR (95% CI)	P trend	Model 3	P	OR (95% CI)	P trend
Q1 Q2 Q3 Q4	Ref 0.98 0.24 0.05	Ref 1.01(0.47,2.17) 1.52(0.74,3.13) 2.25(0.99,5.15)	**0.05**	Q1 Q2 Q3 Q4	Ref 0.21 0.30 0.05	Ref 1.42(0.81,2.47) 1.49(0.68,3.29) 2.11(0.99,4.51)	0.10
Subclinical hyperthyroidism				Subclinical hyperthyroidism			
Model 1	P	OR (95% CI)	P trend	Model 1	P	OR (95% CI)	P trend
Q1 ** Q2** Q3 Q4	Ref **0.04** 0.25 0.67	Ref **0.49(0.25,0.97)** 0.66(0.32,1.36) 1.17(0.55,2.47)	0.82	Q1 **Q2** Q3 Q4	Ref **0.01** 0.85 0.54	Ref **0.29(0.12,0.70)** 1.07(0.51,2.26) 0.84(0.47,1.50)	0.76
Model 2	P	OR (95% CI)	P trend	Model 2	P	OR (95% CI)	P trend
Q1 Q2 Q3 Q4	Ref 0.12 0.48 0.56	Ref 0.55(0.26,1.16) 0.77(0.37,1.61) 1.29(0.54,3.09)	0.55	Q1 **Q2** Q3 Q4	Ref **0.03** 0.58 0.75	Ref **0.33(0.12,0.90)** 1.24(0.57,2.68) 0.88(0.41,1.92)	0.52
Model 3	P	OR (95% CI)	P trend	Model 3	P	OR (95% CI)	P trend
Q1 Q2 Q3 Q4	Ref 0.08 0.36 0.55	Ref 0.53(0.25,1.09) 0.71(0.33,1.53) 1.31(0.53,3.26)	0.65	Q1 **Q2** Q3 Q4	Ref **0.02** 0.85 0.59	Ref **0.30(0.11,0.82)** 1.08(0.47,2.47) 0.81(0.36,1.80)	0.74
Overt hypothyroidism				Overt hypothyroidism			
Model 1	P	OR (95% CI)	P trend	Model 1	P	OR (95% CI)	P trend
Q1 Q2 ** Q3** Q4	Ref 0.09 **0.03** 0.50	Ref 1.83(0.91,3.69) **2.04(1.08,3.85)** 1.19(0.71,1.99)	0.15	Q1 **Q2** ** Q3** ** Q4**	Ref **0.001** **0.004** **0.005**	Ref **2.75(1.51,5.00)** **2.92(1.42,6.01)** **2.33(1.31,4.15)**	**0.001**
Model 2	P	OR (95% CI)	P trend	Model 2	P	OR (95% CI)	P trend
Q1 Q2 Q3 Q4	Ref 0.27 0.20 0.88	Ref 1.53(0.71,3.30) 1.61(0.77,3.38) 0.95(0.52,1.76)	0.88	Q1 **Q2** ** Q3** Q4	Ref **0.01** **0.04** 0.06	Ref **2.39(1.20,4.77)** **2.43(1.06,5.53)** 2.00(0.98,4.07)	0.06
Model 3	P	OR (95% CI)	P trend	Model 3	P	OR (95% CI)	P trend
Q1 Q2 Q3 Q4	Ref 0.28 0.20 0.90	Ref 1.49(0.71,3.15) 1.58(0.77,3.26) 0.96(0.47,1.94)	0.79	Q1 **Q2** **Q3** **Q4**	Ref **0.02** **0.03** **0.05**	Ref **2.26(1.14,4.48)** **2.46(1.08,5.58)** **2.05(1.02,4.16)**	**0.04**
Overt hyperthyroidism				Overt hyperthyroidism			
Model 1	P	OR (95% CI)	P trend	Model 1	P	OR (95% CI)	P trend
Q1 Q2 Q3 Q4	Ref 0.46 0.49 0.20	Ref 1.75(0.39, 7.88) 1.74(0.35, 8.81) 2.74(0.57,13.13)	0.24	Q1 Q2 Q3 Q4	Ref 0.95 0.85 0.12	Ref 1.04(0.24, 4.45) 0.85(0.14, 5.06) 2.85(0.76,10.62	0.22
Model 2	P	OR (95% CI)	P trend	Model 2	P	OR (95% CI)	P trend
Q1 Q2 Q3 Q4	Ref 0.28 0.14 0.07	Ref 2.49(0.46,13.57) 2.95(0.68,12.77) 4.21(0.86,20.57)	**0.05**	Q1 Q2 Q3 Q4	Ref 0.72 0.84 0.07	Ref 1.33(0.27, 6.44) 1.18(0.23, 6.10) 3.58(0.92,13.93	0.1
Model 3	P	OR (95% CI)	P trend	Model 3	P	OR (95% CI)	P trend
Q1 Q2 Q3 Q4	Ref 0.35 0.20 0.14	Ref 2.23(0.39,12.74) 2.69(0.57,12.71) 3.59(0.65,19.87)	0.1	Q1 Q2 Q3 Q4	Ref 0.72 0.84 0.07	Ref 1.26(0.25, 6.37) 1.13(0.20, 6.42) 3.04(0.68,13.52	0.17
PTPOAb				PTPOAb			
Model 1	P	OR (95% CI)	P trend	Model 1	P	OR (95% CI)	P trend
Q1 ** Q2** ** Q3** ** Q4**	Ref **0.01** **<0.001** **<0.001**	Ref **1.52(1.10,2.10)** **1.85(1.38,2.47)** **1.75(1.31,2.35)**	**<0.0001**	Q1 **Q2** ** Q3** ** Q4**	Ref **<0.001** **0.001** **<0.001**	Ref **1.58(1.23,2.03)** **1.72(1.27,2.34)** **1.73(1.31,2.27)**	**<0.001**
Model 2	P	OR (95% CI)	P trend	Model 2	P	OR (95% CI)	P trend
Q1 ** Q2** ** Q3** ** Q4**	Ref **0.05** **0.002** **0.004**	Ref **1.52(1.01,2.29)** **1.87(1.31,2.68)** **1.77(1.22,2.56)**	**<0.001**	Q1 **Q2** ** Q3** ** Q4**	Ref **0.003** **0.002** **<0.001**	Ref **1.60(1.20,2.13)** **1.79(1.28,2.48)** **1.93(1.39,2.70)**	**<0.001**
Model 3	P	OR (95% CI)	P trend	Model 3	P	OR (95% CI)	P trend
Q1 Q2 ** Q3** ** Q4**	Ref 0.07 **0.01** **0.03**	Ref 1.53(0.95,2.46) **1.86(1.24,2.80)** **1.71(1.07,2.73)**	**0.01**	Q1 Q2 **Q3** ** Q4**	Ref **0.01** **0.01** **0.01**	Ref **1.61(1.16,2.25)** **1.81(1.27,2.60)** **1.91(1.29,2.83)**	**0.002**
PTGAb				PTGAb			
Model 1	P	OR (95% CI)	P trend	Model 1	P	OR (95% CI)	P trend
Q1 Q2 Q3 ** Q4**	Ref 0.48 0.20 **<0.001**	Ref 1.14(0.78,1.68) 1.42(0.82,2.46) **2.29(1.56,3.38)**	**<0.001**	Q1 Q2 Q3 **Q4**	Ref 0.25 0.47 **0.001**	Ref 1.30(0.83,2.05) 1.23(0.69,2.22) **2.03(1.36,3.01)**	**0.01**
Model 2	P	OR (95% CI)	P trend	Model 2	P	OR (95% CI)	P trend
Q1 Q2 Q3 ** Q4**	Ref 0.47 0.19 **<0.001**	Ref 1.15(0.77,1.71) 1.49(0.81,2.74) **2.58(1.59,4.20)**	**0.001**	Q1 Q2 Q3 **Q4**	Ref 0.24 0.38 **<0.001**	Ref 1.31(0.82,2.10) 1.30(0.70,2.40) **2.40(1.50,3.84)**	**0.004**
Model 3	P	OR (95% CI)	P trend	Model 3	P	OR (95% CI)	P trend
Q1 Q2 Q3 ** Q4**	Ref 0.51 0.24 **0.002**	Ref 1.14(0.74,1.74) 1.44(0.74,2.81) **2.44(1.53,3.90)**	**0.003**	Q1 Q2 Q3 **Q4**	Ref 0.29 0.47 **0.005**	Ref 1.29(0.77,2.17) 1.25(0.64,2.44) **2.20(1.38,3.51)**	**0.01**

Ref, Reference; OR, Odds ratio;CI, Confidence interval.

The numbers in bold are statistically significant. PTPOAb means the division by the TPOAb positive range (34 IU/mL); PTGAb means the division by the TGAb positive range (4 IU/mL).

For example, individuals in the highest phenotypic age quartile (Q4) showed an elevated odds of subclinical hypothyroidism compared to Q1 (OR = 2.11, 95% CI: 0.99–4.51; P for trend = 0.10). While the confidence interval marginally included 1 and the trend test was not statistically significant, the direction of the association was consistent with findings from the spline analysis.

Similarly, for overt hypothyroidism, the odds ratios in higher phenotypic age quartiles were elevated (Q4 vs Q1: OR = 2.05, 95% CI: 1.02–4.16; P for trend = 0.04), suggesting a statistically significant association.

For thyroid autoimmunity markers, the associations were more robust. The odds of TPOAb positivity increased steadily across phenotypic age quartiles, reaching OR = 1.91 (95% CI: 1.29–2.83) in Q4. Likewise, TGAb positivity showed a significant association with phenotypic age (OR = 2.20, 95% CI: 1.38–3.51 in Q4). These associations remained consistent in the sensitivity analysis (**Supplementary Table S5**), underscoring the robustness of the findings.

### Subgroup analysis

Subgroup analysis were conducted to evaluate the consistency of associations between aging metrics and thyroid dysfunction across different demographic and clinical subgroups (**Supplementary Figures S4**, **S5**). The results were largely consistent, with no substantial interactions altering the primary findings. These subgroup findings, together with additional sensitivity analysis, confirmed the robustness and reliability of the observed relationships.

### Mediation analysis of phenotypic age components

**Figure 3:**
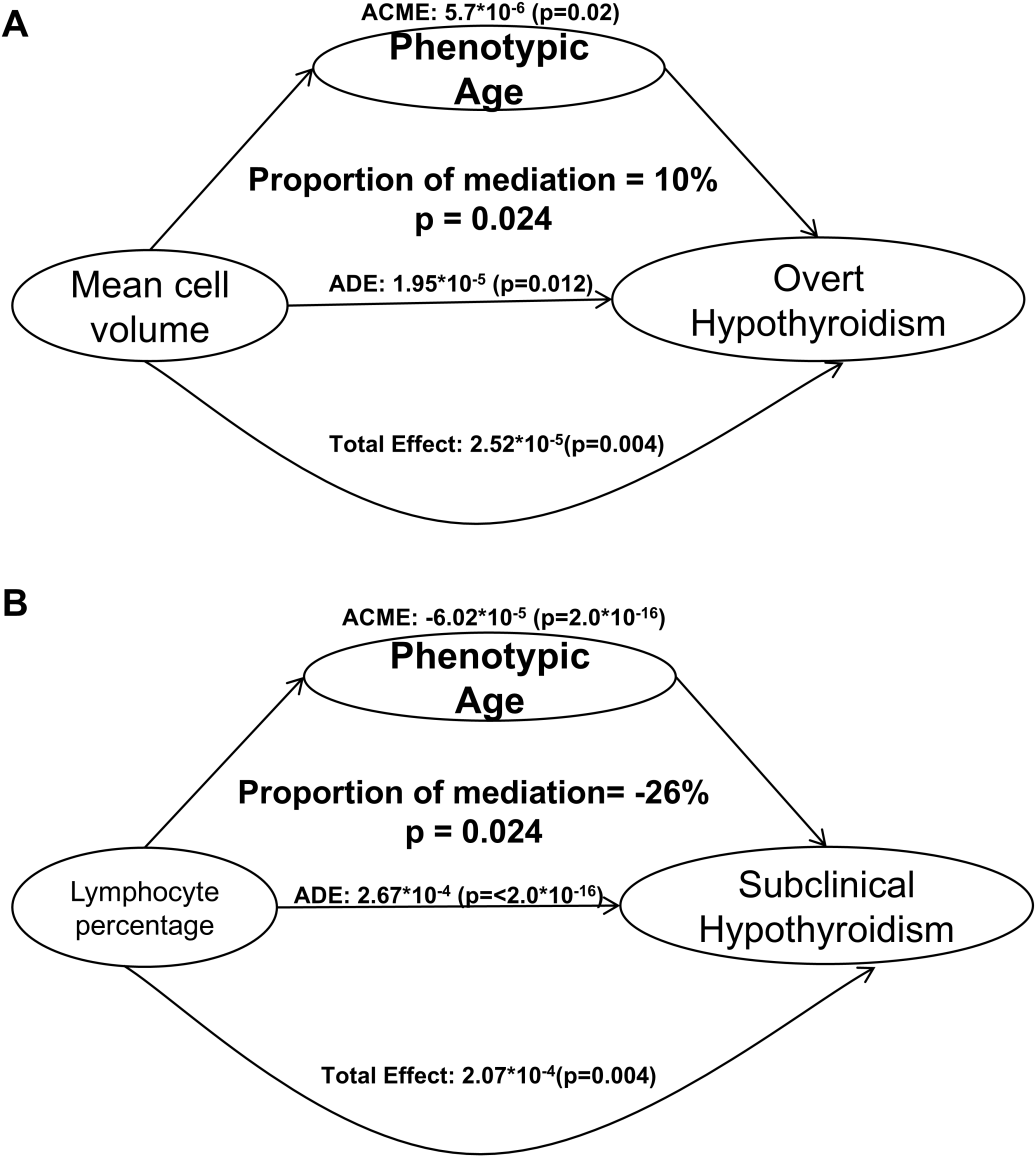
Mediation effects of phenotypic age components on the associations between phenotypic age and thyroid dysfunction. **(A)** Mediation analysis for overt hypothyroidism. **(B)** Mediation analysis for subclinical hypothyroidism. Ten components of phenotypic age were evaluated as potential mediators: albumin, creatinine, glucose, C-reactive protein, lymphocyte percentage, mean cell volume, red cell distribution width, alkaline phosphatase, white blood cell count, and chronological age. Models were adjusted for sex, race/ethnicity, education level, smoking status, alcohol use, body mass index, and comorbidities. ACME, Average Causal Mediation Effect; ADE, Average Direct Effect.

Mediation analysis evaluated whether components of phenotypic age contributed to thyroid dysfunction (**Figure 3**). Mean cell volume (MCV) mediated 10% of the association between phenotypic age and overt hypothyroidism, while lymphocyte percentage (L%) demonstrated a negative mediation effect (–26%) between phenotypic age and subclinical hypothyroidism. These findings suggest that hematological and immune factors partially explain how biological aging contributes to thyroid dysfunction.

## Discussion

This study comprehensively assessed the associations between chronological age, phenotypic age, and thyroid function parameters using a nationally representative dataset. While both aging metrics were linked to thyroid hormone alterations and autoimmune markers, phenotypic age consistently exhibited stronger and more linear associations—particularly with FT3 levels, PTGAb, overt hyperthyroidism, and subclinical hypothyroidism. These findings highlight phenotypic age as a more biologically grounded aging measure, capable of capturing multisystem physiological decline with implications for endocrine health.

The monotonic decline of FT3, in contrast to the U-shaped trends of TSH and FT4, is primarily attributed to changes in T3 production, metabolic demand, and peripheral adaptation. Most T3 is generated by peripheral conversion of T4 in the liver, kidneys, and skeletal muscles, while only a small fraction is directly secreted by the thyroid (27, 28). Aging reduces the activity of iodothyronine deiodinase 1 (DIO1), which converts T4 to T3, and increases DIO3 activity, which inactivates T3 by converting it to reverse T3 (rT3) (29, 30). This imbalance leads to lower FT3 levels in older adults, as demonstrated by studies in both humans and aged mice (10, 13, 18, 31). To compensate, the thyroid increases TSH secretion to elevate T4 levels. However, peripheral tissues may not efficiently convert T4 to T3, prioritizing stable T4 levels to maintain a consistent hormonal reserve. T3 is the biologically active thyroid hormone, with 10- to 30-fold greater receptor affinity than T4 (32, 33). Elevated FT3 levels could pose oxidative stress and metabolic risks in older adults, explaining the physiological preference for maintaining T4 over FT3. Additionally, low FT3 levels are more prone to measurement errors, potentially exaggerating the observed decline (33).

Phenotypic age, as a composite score encompassing chronological age and key clinical parameters, serves as a robust indicator of biological aging across multiple physiological systems (26). This integrated metric moves beyond the linear progression of chronological time, capturing the dynamic interplay of molecular and cellular processes that drive age-related functional decline. Consequently, it offers a more nuanced reflection of an individual’s intrinsic aging trajectory and their vulnerability to age-associated diseases. Compared to chronological age, our findings demonstrate that phenotypic age provides a more detailed reflection of biological aging, with its components individually and collectively linked to thyroid dysfunction in previous studies.

In particular, liver and kidney function can influence thyroid hormone metabolism in multiple ways beyond peripheral T4-to-T3 conversion (29, 31). Albumin, for instance, binds circulating thyroid hormones and modulates their bioavailability (34). Additionally, elevated ALP levels are associated with hyperthyroidism due to increased bone metabolism and hormone-induced cholestasis (35, 36). Serum CR levels vary with thyroid function, being lower in hyperthyroidism (37) and higher in hypothyroidism (38). Studies have shown significant correlations between TSH and CR levels (38), with machine learning models identifying CR as a key parameter in both hyperthyroidism and hypothyroidism models (39).

Thyroid hormones affect hematological parameters both directly, by stimulating erythrocyte precursors, and indirectly, by increasing erythropoietin synthesis (40). Thyroid dysfunction is often associated with MCV, RDW, and WBC counts (41). Autoimmune thyroid disease (AITD), a common comorbidity of pernicious anemia (42) that involves immune dysregulation characterized by lymphocytic infiltration and abnormal T and B cell responses, particularly Hashimoto’s thyroiditis, further underscores the link between thyroid dysfunction and hematological markers (43–45). Thyroid hormones play a critical role in glucose homeostasis by regulating pancreatic β-cell function and glucose metabolism in key organs, including the liver, skeletal muscle, and adipose tissue (46, 47). The frequent co-occurrence of thyroid disorders and diabetes mellitus highlights the interplay between these systems. Overt hypothyroidism is associated with elevated CRP levels (48, 49). In essence, the comprehensive nature of these nine clinical biomarkers allows phenotypic age to capture a broad spectrum of physiological dysregulations that are intimately linked to thyroid hormone synthesis, metabolism, action, and the pathogenesis of thyroid disorders.

To further investigate the biological mechanisms through which phenotypic age influences thyroid dysfunction, we conducted a mediation analysis using its ten component markers. Notably, MCV mediated 10% of the association between phenotypic age and overt hypothyroidism. MCV tends to increase with age and is frequently elevated in hypothyroidism, reflecting macrocytic anemia caused by reduced thyroid hormone–stimulated erythropoiesis and possible nutrient deficiencies (40, 41). In contrast, Lymphocyte Percentage (L%) demonstrated a−26% mediation effect in the relationship between Phenotypic Age and subclinical hypothyroidism. While early autoimmune thyroiditis may present with elevated lymphocyte levels (43, 45), biological aging is often accompanied by immunosenescence, characterized by lymphocyte decline and reduced immune responsiveness (50). This may suppress the autoimmune activation seen in subclinical hypothyroidism, explaining the observed inverse mediation.

By integrating diverse physiological markers, phenotypic age offers a valuable framework for understanding the complex interplay between thyroid dysfunction and systemic aging, while emerging clinical trials on reversing biological aging present promising opportunities for more personalized management and preventive interventions for thyroid dysfunction diseases in the future (51).

Despite the strengths of our study, several limitations should be acknowledged. First, the cross-sectional design of the NHANES dataset prevents the establishment of causal relationships and does not capture time-dependent trajectories. Second, all biomarkers and thyroid function indicators were assessed at a single time point, which may reflect transient fluctuations rather than sustained physiological states, potentially impacting classification reliability. Third, the mediation effects of MCV and L%, although statistically significant, were modest in magnitude, suggesting the involvement of other unmeasured biological or environmental mediators. Fourth, phenotypic age components are inherently interrelated, and their observed effects may reflect overlapping biological processes rather than independent pathways. Fifth, the use of a U.S.-based population limits the generalizability of our findings to other ethnic or geographic populations. Sixth, the definitions of subclinical thyroid dysfunction may lead to misclassification. For example, subclinical hypothyroidism, defined as TSH > 5.6 mU/L with normal FT4, does not account for age-related increases in TSH reference ranges. Similarly, subclinical hyperthyroidism may overlap with non-thyroidal illness (NTI), which is common among older adults and may confound interpretation. Lastly, future research should incorporate more comprehensive aging-related indicators—such as genomic and epigenomic markers, sleep quality, and psychological stress levels—to further validate and expand the biological pathways proposed in this study. Longitudinal data from future NHANES cycles or other prospective cohorts are also needed to assess the temporal stability of these associations and to evaluate their applicability across diverse populations, including Asian and African cohorts.

## Data Availability

The datasets presented in this study can be found in online repositories. The names of the repository/repositories and accession number(s) can be found below: https://wwwn.cdc.gov/nchs/nhanes/Default.aspx.

## Supplementary material

Supplementary Figure 1 Flowchart of participant selection in the study, NHANES 2007–2012. The dotted lines represent participant exclusion.

Supplementary Figure 2 The relationship between chronological age and phenotypic age is depicted using Restricted Cubic Splines (RCSs). P for overall less than 0.05 suggests a significant association between X and Y. The "P for Non-linearity" value assesses whether the relationship is nonlinear. If this value is less than 0.05, it supports a nonlinear relationship between X and Y. Conversely, if "P for Non-linearity" is 0.05 or higher, it indicates a linear relationship between the two variables.

Supplementary Figure 3 Population density plots for chronological age, phenotypic age, and age gap. Panels **(A-C)** show density plots illustrating the distribution of the study population by chronological age **(A)**, phenotypic age **(B)**, and age gap **(C)**. The x-axis represents age, phenotypic age, or age gap in years, and the y-axis represents the fraction of the population (density).

Supplementary Figure 4 The effect of chronological age **(A, C)** and phenotypic age **(B, D)** on the prevalence of overt hypothyroidism and subclinical hypothyroidism in subgroups. Abbreviations: HR, hazard ratio.

Supplementary Figure 5 The effect of chronological age **(A)** and phenotypic age **(B)** on the prevalence of subclinical hyperthyroidism in subgroups. Abbreviations: HR, hazard ratio.

Supplementary Table 1 Missing counts.
