# Supplementary figures and images for: Assessing thyroid health: phenotypic age compared to chronological age
Source: Front Endocrinol (Lausanne). 2025 Jul 4;16:1594139. doi: 10.3389/fendo.2025.1594139 (PMC12270862; doi:10.3389/fendo.2025.1594139)

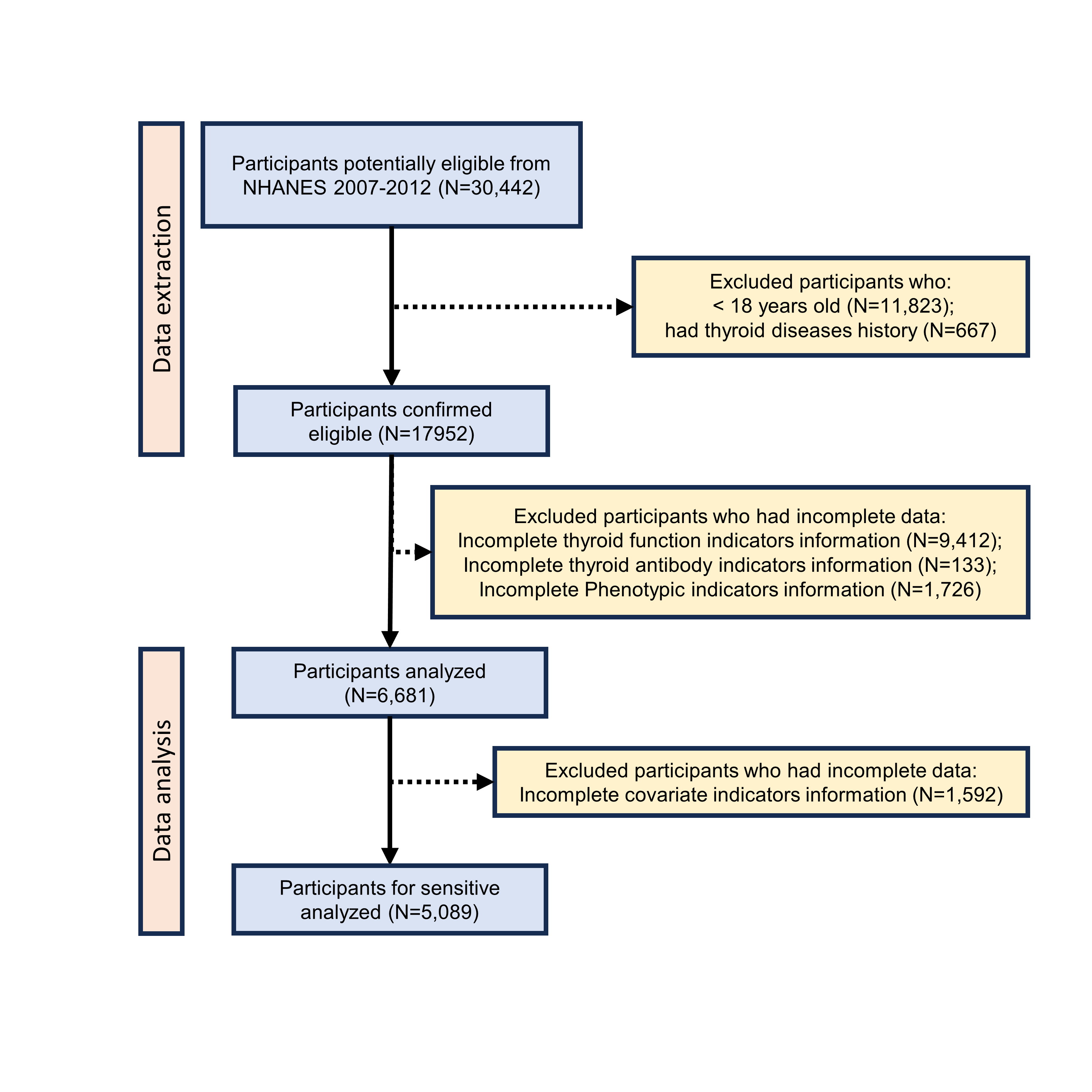

Supplement: Supplementary Figure 1 — Flowchart of participant selection in the study, NHANES 2007–2012. The dotted lines represent participant exclusion. [file Image1.tif]

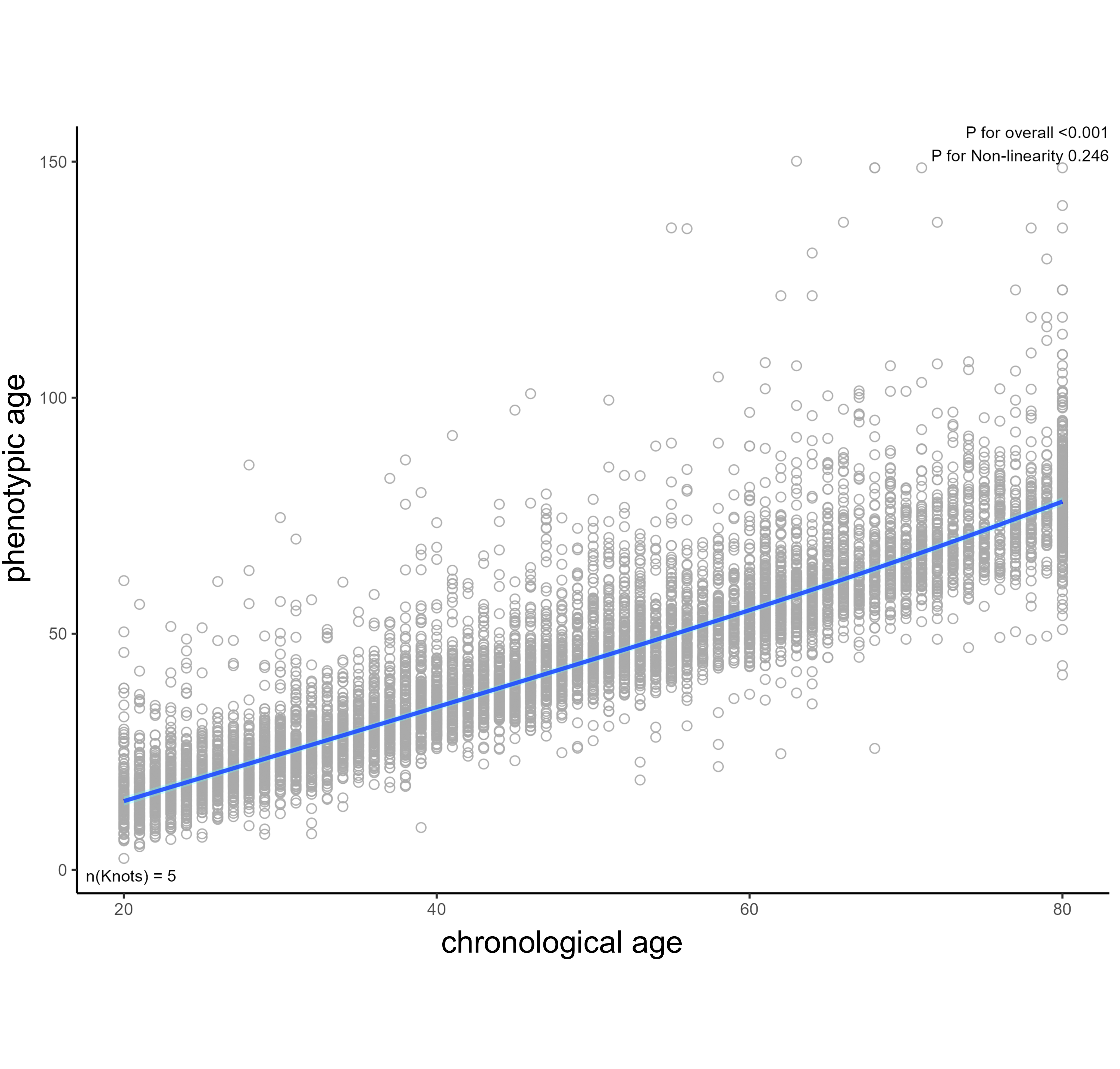

Supplement: Supplementary Figure 2 — The relationship between chronological age and phenotypic age is depicted using Restricted Cubic Splines (RCSs). P for overall less than 0.05 suggests a significant association between X and Y. The "P for Non-linearity" value assesses whether the relationship is nonlinear. If this value is less than 0.05, it supports a nonlinear relationship between X and Y. Conversely, if "P for Non-linearity" is 0.05 or higher, it indicates a linear relationship between the two variables. [file Image2.tif]

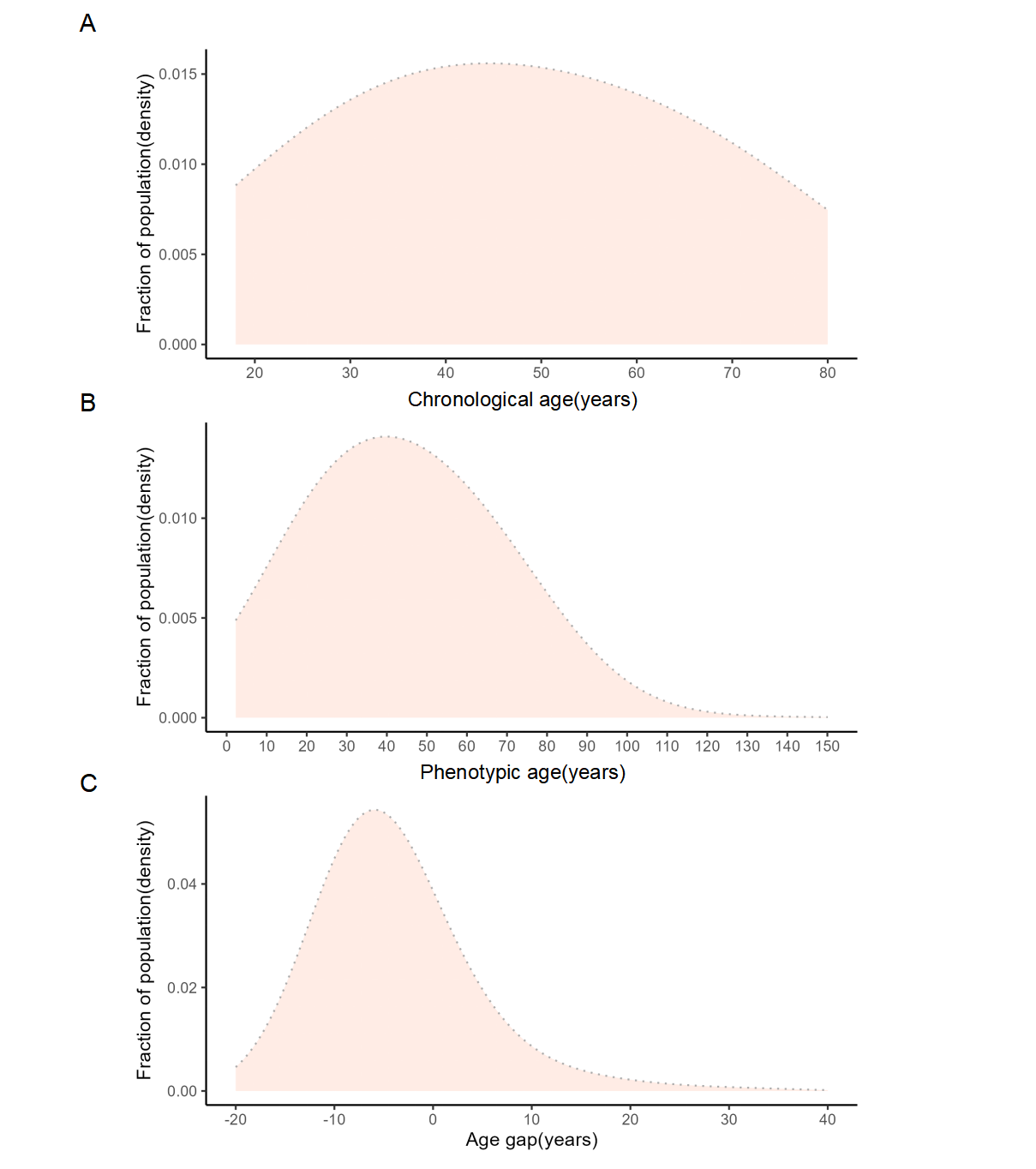

Supplement: Supplementary Figure 3 — Population density plots for chronological age, phenotypic age, and age gap. Panels (A-C) show density plots illustrating the distribution of the study population by chronological age (A), phenotypic age (B), and age gap (C). The x-axis represents age, phenotypic age, or age gap in years, and the y-axis represents the fraction of the population (density). [file Image3.tif]

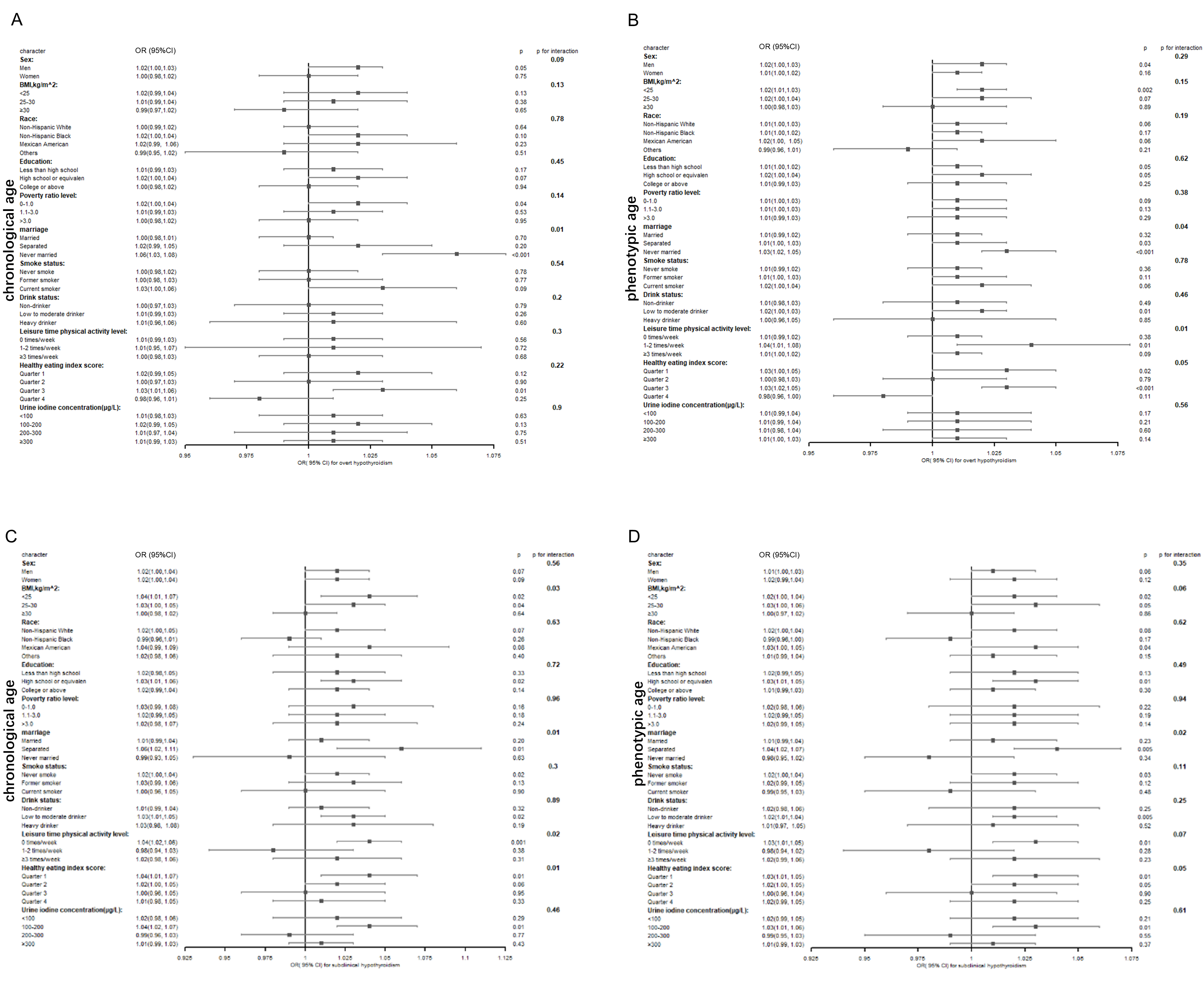

Supplement: Supplementary Figure 4 — The effect of chronological age (A, C) and phenotypic age (B, D) on the prevalence of overt hypothyroidism and subclinical hypothyroidism in subgroups. Abbreviations: HR, hazard ratio. [file Image4.tif]

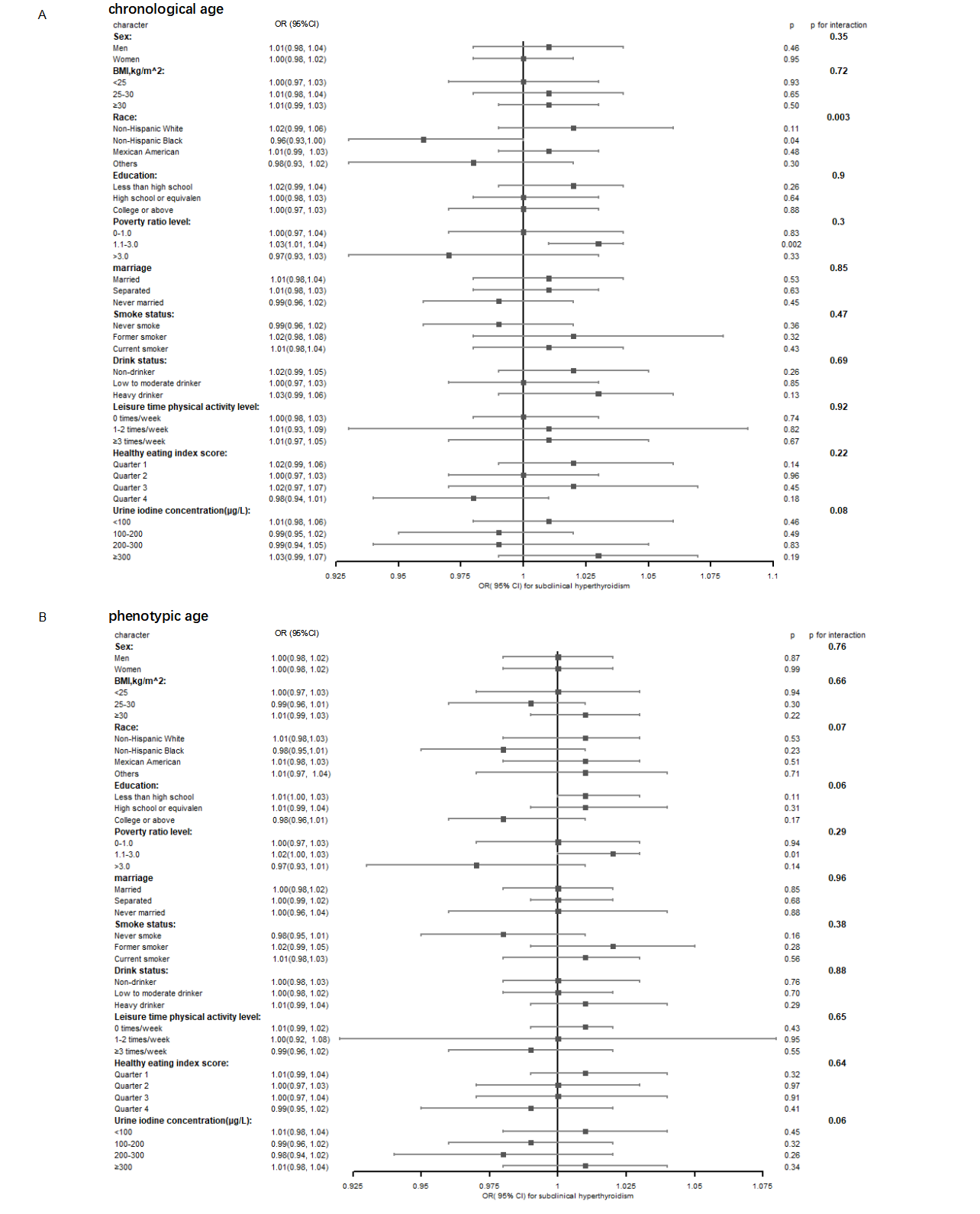

Supplement: Supplementary Figure 5 — The effect of chronological age (A) and phenotypic age (B) on the prevalence of subclinical hyperthyroidism in subgroups. Abbreviations: HR, hazard ratio. [file Image5.tif]
