# Supplementary material for: Assessing thyroid health: phenotypic age compared to chronological age
Source: Front Endocrinol (Lausanne). 2025 Jul 4;16:1594139. doi: 10.3389/fendo.2025.1594139 (PMC12270862; doi:10.3389/fendo.2025.1594139)
Supplement: Supplementary file 7 [file Table2.docx]

Supplement Table 2 Thyroid indicators and prevalence of thyroid diseases based on chronological age quartiles

| Characteristic | Total | Quartile 1 | Quartile 2 | Quartile 3 | Quartile 4 | *P* value |
| --- | --- | --- | --- | --- | --- | --- |
| Thyroid parameters^a^ |  |  |  |  |  |  |
| TSH(mIU/L) | 1.91(1.83 - 2.00) | 1.68(1.60 - 1.77) | 1.82(1.72 - 1.92) | 2.04(1.91 - 2.18) | 2.25(2.16 - 2.34) | < 0.0001 |
| FT4(pmol/L) | 10.02(9.87 - 10.18) | 10.16(9.96 - 10.37) | 9.81(9.66 - 9.96) | 9.87(9.69 - 10.05) | 10.40(10.23 - 10.58) | < 0.0001 |
| FT3(pg/mL) | 3.21(3.19 - 3.23) | 3.37(3.34 - 3.40) | 3.25(3.22 - 3.29) | 3.15(3.13 - 3.17) | 2.97(2.95 - 3.00) | < 0.0001 |
| TT4(ug/dL) | 7.76(7.68 - 7.84) | 7.85(7.75 - 7.96) | 7.65(7.54 - 7.76) | 7.73(7.63 - 7.83) | 7.85(7.73 - 7.97) | < 0.001 |
| TT3(ng/dL) | 114.88(113.48 - 116.27) | 122.35(120.63 - 124.07) | 116.19(114.33 - 118.04) | 112.38(110.66 - 114.10) | 103.80(102.04 - 105.56) | < 0.0001 |
| TPOAb(IU/mL) | 16.60(14.78 - 18.43) | 12.73(9.35 - 16.10) | 18.24(14.12 - 22.37) | 20.03(15.71 - 24.36) | 14.98(10.74 - 19.22) | 0.063 |
| TgAb(IU/mL) | 8.18(6.17 - 10.19) | 3.57(1.49 - 5.64) | 7.01(2.75 - 11.27) | 8.81(4.34 - 13.27) | 17.07(9.54 - 24.60) | 0.0015 |
| Thyroid diseases^b^ |  |  |  |  |  |  |
| Subclinical hypothyroidism | 104(1.58%) | 15(1.16%) | 21(1.30%) | 26(1.77%) | 42(2.47%) | 0.1623 |
| Subclinical hyperthyroidism | 72(0.89%) | 28(1.23%) | 13(0.53%) | 14(0.75%) | 17(1.16%) | 0.1128 |
| Overt hypothyroidism | 161(2.35%) | 25(1.30%) | 40(2.57%) | 57(3.67%) | 39(1.69%) | < 0.001 |
| Overt hyperthyroidism | 16(0.16%) | 2(0.09%) | 6(0.21%) | 2(0.12%) | 6(0.29%) | 0.4691 |
| TPOAb(IU/mL)^b^ |  |  |  |  |  | < 0.001 |
| < 34 | 6085(90.63%) | 1608(93.51%) | 1550(90.45%) | 1427(88.63%) | 1500(89.15%) |  |
| > 34 | 596(9.37%) | 110( 6.49%) | 149( 9.55%) | 168(11.37%) | 169(10.85%) |  |
| TgAb(IU/mL)^b^ |  |  |  |  |  | 0.0017 |
| < 4 | 6298(94.23%) | 1644(95.68%) | 1619(95.09%) | 1510(93.98%) | 1525(90.62%) |  |
| > 4 | 383(5.77%) | 74(4.32%) | 80(4.91%) | 85(6.02%) | 144(9.38%) |  |

Abbreviations: TgAb, Thyroglobulin antibody; TPOAb, Thyroid peroxidase antibody

^a^ presented as mean(95% confidence interval)

^b^ presented as mean(frequency)

PTPOAb means the division by the TPOAb positive range(34 IU/mL); PTgAb means the division by the TgAb positive range(4 IU/mL)
