# Supplementary material for: Assessing thyroid health: phenotypic age compared to chronological age
Source: Front Endocrinol (Lausanne). 2025 Jul 4;16:1594139. doi: 10.3389/fendo.2025.1594139 (PMC12270862; doi:10.3389/fendo.2025.1594139)
Supplement: Supplementary file 8 [file Table3.docx]

Supplement Table 3 Thyroid indicators and prevalence of thyroid diseases based on phenotypic age quartiles

| Characteristic | Total | Quartile 1 | Quartile 2 | Quartile 3 | Quartile 4 | *P* value |
| --- | --- | --- | --- | --- | --- | --- |
| Thyroid parameters^a^ |  |  |  |  |  |  |
| TSH (mIU/L) | 1.91 (1.83 - 2.00) | 1.68 (1.58 - 1.77) | 1.85 (1.74 - 1.95) | 2.04 (1.95 - 2.14) | 2.23 (2.11 - 2.35) | < 0.0001 |
| FT4 (pmol/L) | 10.02 (9.87 - 10.18) | 10.21 (10.02 - 10.40) | 9.73 (9.57 - 9.90) | 9.88 (9.73 - 10.04) | 10.47 (10.27 - 10.66) | < 0.0001 |
| FT3 (pg/mL) | 3.21 (3.19 - 3.23) | 3.37 (3.34 - 3.40) | 3.24 (3.21 - 3.27) | 3.15 (3.13 - 3.17) | 2.97(2.95 - 3.00) | < 0.0001 |
| TT4 (ug/dL) | 7.76 (7.68 - 7.84) | 7.81 (7.70 - 7.91) | 7.64 (7.52 - 7.76) | 7.75 (7.65 - 7.84) | 7.91 (7.78 - 8.05) | < 0.001 |
| TT3 (ng/dL) | 114.88 (113.48 - 116.27) | 121.46 (119.75 - 123.17) | 116.23 (113.8 - 118.67) | 113.83 (112.15 - 115.5) | 102.6 (100.95 - 104.25) | < 0.0001 |
| TPOAb (IU/mL) | 16.60 (14.78 - 18.43) | 11.78 (8.57 - 14.98) | 21.18 (16.82 - 25.53) | 16.48 (13.06 - 19.9) | 16.89 (11.61 - 22.17) | 0.01 |
| TgAb (IU/mL) | 8.18 (6.17 - 10.19) | 5.43 (1.84 - 9.01) | 6.89 (3.04 - 10.74) | 7.96 (3.89 - 12.02) | 15.72 (7.93 - 23.52) | 0.04 |
| Thyroid diseases^b^ |  |  |  |  |  |  |
| Subclinical hypothyroidism | 104(1.58%) | 14(0.98%) | 20(1.4%) | 28(1.83%) | 42(2.52%) | 0.0603 |
| Subclinical hyperthyroidism | 72(0.89%) | 27(1.29%) | 10(0.34%) | 19(1.07%) | 16(0.91%) | 0.0368 |
| Overt hypothyroidism | 161(2.35%) | 17(0.91%) | 46(2.88%) | 54(3.27%) | 44(2.38%) | 0.0018 |
| Overt hyperthyroidism | 16(0.16%) | 4(0.15%) | 4(0.15%) | 2(0.12%) | 6(0.30%) | 0.6125 |
| TPOAb (IU/mL)^b^ |  |  |  |  |  | 0.0012 |
| < 34 | 6085(90.63%) | 1560(93.43%) | 1520(90.01%) | 1505(89.21%) | 1500(89.18%) |  |
| > 34 | 596(9.37%) | 111(6.57%) | 149(9.99%) | 166(10.79%) | 170(10.82%) |  |
| TgAb (IU/mL)^b^ |  |  |  |  |  | 0.023 |
| < 4 | 6298(94.23%) | 1596(95.53%) | 1580(94.26%) | 1589(94.55%) | 1533(91.34%) |  |
| > 4 | 383(5.77%) | 75(4.47%) | 89(5.74%) | 82(5.45%) | 137(8.66%) |  |

Abbreviations: TgAb, Thyroglobulin antibody; TPOAb, Thyroid peroxidase antibody

^a^ presented as mean (95% confidence interval)

^b^ presented as mean (frequency)

PTPOAb means the division by the TPOAb positive range (34 IU/mL); PTgAb means the division by the TgAb positive range (4 IU/mL)
