# Supplementary material for: Assessing thyroid health: phenotypic age compared to chronological age
Source: Front Endocrinol (Lausanne). 2025 Jul 4;16:1594139. doi: 10.3389/fendo.2025.1594139 (PMC12270862; doi:10.3389/fendo.2025.1594139)
Supplement: Supplementary file 9 [file Table4.docx]

Supplement Table 4 Thyroid indicators and prevalence of thyroid diseases based on age gap quartiles

| Characteristic | Total | Quartile 1 | Quartile 2 | Quartile 3 | Quartile 4 | *P* value |
| --- | --- | --- | --- | --- | --- | --- |
| Thyroid parameters^a^ |  |  |  |  |  |  |
| TSH (mIU/L) | 1.91 (1.83 - 2.00) | 1.86 (1.75 - 1.97) | 1.91 (1.80 - 2.01) | 1.95 (1.75 - 2.15) | 1.94 (1.84 - 2.05) | 0.77 |
| FT4 (pmol/L) | 10.02 (9.87 - 10.18) | 10.00 (9.80 - 10.19) | 10.00 (9.84 - 10.15) | 9.98 (9.83 - 10.14) | 10.15 (9.92 - 10.38) | 0.26 |
| FT3 (pg/mL) | 3.21 (3.19 - 3.23) | 3.20 (3.17 - 3.22) | 3.25 (3.22 - 3.27) | 3.25 (3.22 - 3.27) | 3.14 (3.11 - 3.17) | < 0.0001 |
| TT4 (ug/dL) | 7.76 (7.68 - 7.84) | 7.60 (7.49 - 7.71) | 7.68 (7.58 - 7.78) | 7.80 (7.72 - 7.87) | 8.04 (7.90 - 8.18) | < 0.0001 |
| TT3 (ng/dL) | 114.88 (113.48 - 116.27) | 114.60 (112.92 - 116.28) | 115.51 (113.76 - 117.26) | 116.71 (114.69 - 118.73) | 112.16 (110.37 - 113.94) | < 0.001 |
| TPOAb (IU/mL) | 16.60 (14.78 - 18.43) | 15.85 (11.13 - 20.57) | 17.03 (11.25 - 22.82) | 16.66 (11.22 - 22.09) | 17.03 (11.92 - 22.15) | 0.99 |
| TgAb (IU/mL) | 8.18 (6.17 - 10.19) | 9.30 (4.68 - 13.91) | 6.99 (3.13 - 10.85) | 7.73 (4.14 - 11.33) | 8.75 (3.29 - 14.20) | 0.79 |
| Thyroid diseases^b^ |  |  |  |  |  |  |
| Subclinical hypothyroidism | 104(1.58%) | 22(1.66%) | 21(1.10%) | 26(1.78%) | 35(1.85%) | 0.57 |
| Subclinical hyperthyroidism | 72(0.89%) | 22(1.26%) | 13(0.54%) | 19(0.88%) | 18(0.86%) | 0.18 |
| Overt hypothyroidism | 161(2.35%) | 29(1.46%) | 34(2.09%) | 35(2.05%) | 63(4.31%) | 0.01 |
| Overt hyperthyroidism | 16(0.16%) | 3(0.09%) | 2(0.08%) | 5(0.27%) | 6(0.25%) | 0.27 |
| TPOAb (IU/mL)^b^ |  |  |  |  |  | 0.62 |
| < 34 | 6085(90.63%) | 1560(93.43%) | 1520(90.01%) | 1505(89.21%) | 1500(89.18%) |  |
| > 34 | 596(9.37%) | 155(10.06%) | 150( 8.30%) | 147( 9.31%) | 144( 9.91%) |  |
| TgAb (IU/mL)^b^ |  |  |  |  |  | 0.46 |
| < 4 | 6298(94.23%) | 1596(95.53%) | 1580(94.26%) | 1589(94.55%) | 1533(91.34%) |  |
| > 4 | 383(5.77%) | 101(6.60%) | 92(5.08%) | 95(5.24%) | 95(6.19%) |  |

Abbreviations: TgAb, Thyroglobulin antibody; TPOAb, Thyroid peroxidase antibody

^a^ presented as mean (95% confidence interval)

^b^ presented as mean (frequency)

PTPOAb means the division by the TPOAb positive range (34 IU/mL); PTgAb means the division by the TgAb positive range (4 IU/mL)
