# Supplementary material for: Assessing thyroid health: phenotypic age compared to chronological age
Source: Front Endocrinol (Lausanne). 2025 Jul 4;16:1594139. doi: 10.3389/fendo.2025.1594139 (PMC12270862; doi:10.3389/fendo.2025.1594139)
Supplement: Supplementary file 10 [file Table5.docx]

Supplement Table 5 Sensitivity analysis of the relationship between chronological age and phenotypic age based on interquartile range and subclinical hypothyroidism, subclinical hyperthyroidism, overt hypothyroidism, overt hyperthyroidism, and thyroid autoimmunity indicators

|  | Chronological age | | |  |  | Phenotypic age | | |  |
| --- | --- | --- | --- | --- | --- | --- | --- | --- | --- |
|  | P | OR (95% CI) | P trend |  |  | P | OR (95% CI) | P trend |  |
| Subclinical hypothyroidism: |  |  |  |  | Subclinical hypothyroidism: |  |  |  |  |
| Model 1  Q1  Q2  Q3  Q4 | Ref  0.43  0.04  <0.001 | Ref  1.36(0.62,2.99)  1.89(1.02,3.51)  2.87(1.64,5.04) | 0.01 |  | Model 1  Q1  Q2  Q3  Q4 | Ref  0.05  0.24  0.01 | Ref  1.81(1.00,3.25) 1.64(0.71,3.83)  3.02(1.39,6.55) | 0.03 |  |
| Model 2  Q1  Q2  Q3  Q4 | Ref  0.65  0.18  0.01 | Ref  1.24(0.46,3.35)  1.76(0.75,4.12)  2.42(1.22,4.79) | 0.04 |  | Model 2  Q1  Q2  Q3  Q4 | Ref  0.10  0.33  0.05 | Ref  1.69(0.90,3.15) 1.58(0.60,4.15)  2.69(1.00,7.24) | 0.13 |  |
| Model 3  Q1  Q2  Q3  Q4 | Ref  0.95  0.18  0.03 | Ref  1.04(0.31,3.50)  1.82(0.70,4.74)  3.09(1.12,8.47) | 0.05 |  | Model 3  Q1  Q2  Q3  Q4 | Ref  0.51  0.47  0.09 | Ref  1.23(0.60,2.56) 1.43(0.45,4.53)  2.56(0.82,8.00) | 0.13 |  |
| Subclinical hyperthyroidism: |  |  |  |  | Subclinical hyperthyroidism: |  |  |  |  |
| Model 1  Q1  Q2  Q3  Q4 | Ref  0.64  0.55  0.39 | Ref  0.81(0.33,1.99)  1.29(0.54,3.10)  1.40(0.64,3.10) | 0.26 |  | Model 1  Q1  Q2  Q3  Q4 | Ref  0.09  0.40  0.80 | Ref  0.38(0.12,1.16) 1.46(0.59,3.62)  0.90(0.39,2.09) | 0.45 |  |
| Model 2  Q1  Q2  Q3  Q4 | Ref  0.53  0.66  0.58 | Ref  0.74(0.28,1.97)  1.21(0.50,2.94)  1.24(0.55,2.76) | 0.33 |  | Model 2  Q1  Q2  Q3  Q4 | Ref  0.10  0.55  0.53 | Ref  0.35(0.10,1.26) 1.31(0.51,3.34)  0.74(0.27,1.98) | 0.70 |  |
| Model 3  Q1  Q2  Q3  Q4 | Ref  0.45  0.79  0.50 | Ref  0.70(0.23,2.10)  1.14(0.36,3.64)  1.46(0.41,5.17) | 0.38 |  | Model 3  Q1  Q2  Q3  Q4 | Ref  0.10  0.74  0.58 | Ref  0.32(0.08,1.36) 1.18(0.36,3.87)  0.72(0.19,2.79) | 0.81 |  |
| Overt hypothyroidism: |  |  |  |  | Overt hypothyroidism: |  |  |  |  |
| Model 1  Q1  Q2  Q3  Q4 | Ref  0.35  0.14  0.97 | Ref  1.50(0.63,3.56)  1.84(0.81,4.19)  1.01(0.58,1.76) | 0.36 |  | Model 1  Q1  Q2  Q3  Q4 | Ref  0.001  0.01  0.01 | Ref  3.26(1.66,6.39) 2.80(1.28,6.12)  2.48(1.24,4.99) | 0.001 |  |
| Model 2  Q1  Q2  Q3  Q4 | Ref  0.58  0.34  0.83 | Ref  1.30(0.48,3.49)  1.57(0.60,4.15)  0.92(0.44,1.94) | 0.74 |  | Model 2  Q1  Q2  Q3  Q4 | Ref  0.01  0.05  0.06 | Ref  2.96(1.36,6.44) 2.44(1.00,5.95)  2.30(0.95,5.58) | 0.06 |  |
| Model 3  Q1  Q2  Q3  Q4 | Ref  0.58  0.33  0.96 | Ref  1.31(0.43,4.01)  1.65(0.53,5.15)  0.98(0.36,2.68) | 0.63 |  | Model 3  Q1  Q2  Q3  Q4 | Ref  0.02  0.05  0.09 | Ref  2.97(1.31,6.77)  2.72(0.99,7.49) 2.42(0.82,7.16) | 0.04 |  |
| Overt hyperthyroidism: |  |  |  |  | Overt hyperthyroidism: |  |  |  |  |
| Model 1  Q1  Q2  Q3  Q4 | Ref  0.80  0.52  0.38 | Ref  0.74(0.06, 8.96)  1.92(0.24,15.33) 2.31(0.33,15.98) | 0.29 |  | Model 1  Q1  Q2  Q3  Q4 | Ref  0.80  0.50  0.36 | Ref  0.74(0.06, 9.01)  2.03(0.25,16.6)  2.39(0.35,16.45) | 0.26 |  |
| Model 2  Q1  Q2  Q3  Q4 | Ref  0.86  0.06  0.04 | Ref  1.22(0.12,12.63)  3.89(0.95,15.94)  5.09(1.07,24.18) | 0.01 |  | Model 2  Q1  Q2  Q3  Q4 | Ref  0.89  0.06  0.06 | Ref  1.17(0.11,12.92)  3.79(0.96,15.01)  5.12(0.91,28.76) | 0.02 |  |
| Model 3  Q1  Q2  Q3  Q4 | Ref  0.86  0.09  0.08 | Ref  1.23(0.08,18.38)  4.38(0.73,26.38)  5.38(0.75,38.74) | 0.03 |  | Model 3  Q1  Q2  Q3  Q4 | Ref  0.89  0.08  0.13 | Ref  1.17(0.08,17.94)  4.32(0.76,24.49)  4.53(0.54,37.92) | 0.05 |  |
| PTPOAb (IU/mL): |  |  |  |  | PTPOAb (IU/mL): |  |  |  |  |
| Model 1  Q1  Q2  Q3  Q4 | Ref  0.01  <0.001  0.004 | Ref  1.52(1.10,2.10)  1.79(1.31,2.44)  1.58(1.17,2.12) | 0.001 |  | Model 1  Q1  Q2  Q3  Q4 | Ref  0.001  0.01  0.004 | Ref  1.64(1.23,2.19)  1.73(1.19,2.51)  1.65(1.19,2.28) | 0.002 |  |
| Model 2  Q1  Q2  Q3  Q4 | Ref  0.04  0.01  0.02 | Ref  1.49(1.01,2.19)  1.76(1.19,2.60)  1.60(1.11,2.32) | 0.01 |  | Model 2  Q1  Q2  Q3  Q4 | Ref  0.004  0.01  0.004 | Ref  1.67(1.20,2.33)  1.80(1.17,2.78)  1.92(1.27,2.91) | 0.004 |  |
| Model 3  Q1  Q2  Q3  Q4 | Ref  0.07  0.02  0.06 | Ref  1.49(0.95,2.32)  1.74(1.12,2.70)  1.51(0.98,2.34) | 0.02 |  | Model 3  Q1  Q2  Q3  Q4 | Ref  0.01  0.02  0.02 | Ref  1.68(1.16,2.45)  1.81(1.13,2.91)  1.90(1.17,3.11) | 0.01 |  |
| PTgAb (IU/mL): |  |  |  |  | PTgAb (IU/mL): |  |  |  |  |
| Model 1  Q1  Q2  Q3  Q4 | Ref  0.57  0.11  <0.001 | Ref  1.15(0.70,1.91)  1.61(0.90,2.89)  2.34(1.51,3.62) | <0.001 |  | Model 1  Q1  Q2  Q3  Q4 | Ref  0.06  0.14  <0.0001 | Ref  1.60(0.98,2.62) 1.58(0.85,2.92)  2.47(1.67,3.66) | <0.001 |  |
| Model 2  Q1  Q2  Q3  Q4 | Ref  0.69  0.15  0.002 | Ref  1.11(0.65,1.88)  1.58(0.83,3.01)  2.47(1.46,4.19) | 0.002 |  | Model 2  Q1  Q2  Q3  Q4 | Ref  0.07  0.12  <0.001 | Ref  1.61(0.96,2.69)  1.64(0.86,3.14)  2.92(1.85,4.63) | <0.001 |  |
| Model 3  Q1  Q2  Q3  Q4 | Ref  0.70  0.21  0.01 | Ref  1.10(0.62,1.97)  1.53(0.75,3.16)  2.24(1.31,3.85) | 0.01 |  | Model 3  Q1  Q2  Q3  Q4 | Ref  0.10  0.18  0.001 | Ref  1.59(0.89,2.84)  1.60(0.77,3.32)  2.68(1.67,4.29) | 0.002 |  |
